# Health and health risk behaviour of adolescents—Differences according to family structure. Results of the German KiGGS cohort study

**DOI:** 10.1371/journal.pone.0192968

**Published:** 2018-03-07

**Authors:** Petra Rattay, Elena von der Lippe, Elvira Mauz, Felicitas Richter, Heike Hölling, Cornelia Lange, Thomas Lampert

**Affiliations:** Department of Epidemiology and Health Monitoring, Robert Koch Institute, Berlin, Germany; University Children’s Hospital Tuebingen, GERMANY

## Abstract

**Objective:**

This study’s aim was to investigate the association between family structure and different health-related outcomes in adolescence (self-rated health, emotional and behavioural problems, health-related quality of life, regular smoking, and heavy episodic drinking). Furthermore, we analysed the extent to which socio-economic status, family cohesion and the pre-transition health status explain family structure-related health disparities.

**Methods:**

We used longitudinal data from the first two waves of the German KiGGS cohort study carried out by the Robert Koch Institute (baseline: 2003–2006, follow-up: 2009–2012). The sample comprised 4,692 respondents aged 11 to 17 years. Using data from both waves, effects of family structure on health status at follow-up were calculated applying linear and logistic regression models.

**Results:**

We found that adolescents continuously living with both birth parents were in good health. Adolescents whose parents separated after the baseline survey, reported poorer health and were more likely to smoke. The transition from stepfamily to single parent family was also associated with a higher risk of regular smoking. Lower health-related quality of life as well as higher scores for emotional and behavioural problems occurred in almost all non-nuclear family structures, although not all effects were statistically significant. No significant effects of family structure on heavy episodic drinking were found. While family cohesion mediated the effects of family structure on adolescents’ health, the mediating effect of socio-economic status was small. After controlling for pre-transition health, the effects were even lower.

**Conclusions:**

Because the direct effects of family structure on adolescents’ health were small and family cohesion was found to be an important mediator in the association between family structure and adolescents’ health, prevention programmes and interventions should be directed towards the parent–adolescent relationship rather than just the family structure, in order to minimize the psychosocial stress of adolescents during the period of family transition.

## Introduction

The diversity of family arrangements is rising in developed countries. In Germany in 2013, 18% of the 13 million children younger than age 18 were living in single parent families [1]. Nine out of ten single parents were mothers. Single fathers lived more often with adolescents than with children of younger age [1]. Official data on the number of children and adolescents living in stepfamilies were not available for Germany. Estimates from scientific studies on the proportion of children and adolescents living in stepfamilies varied between 6.0% [2] and 10.9% [3].

The separation of birth parents is considered a major critical life event in childhood and youth [4]. This is particularly the case when children have experienced strong conflicts in their family. Furthermore, the parents’ separation is not a circumscribed event, but can be accompanied by changes in the social network and economic conditions of young people. This may include, for example, a loss of contact with the non-resident parent or with friends because of moving to another neighbourhood or changing schools. Additionally, less favourable time and economic resources in a single parent family can be aftereffects of the parents’ separation. In Germany, single parent families in particular are affected by poverty. Data show that 39% of these families received basic security benefits (Book II of the Social Code) in 2012 [1]. However, the separation of parents can sometimes bring some advantages for the child’s development—mostly owing to the reduction or end of a highly conflicted partnership of the birth parents [4].

If the custodial parent starts living with a new partner, on the one hand, this can be associated with an improvement of the social and financial resources of the family, so that children benefit from the new partner’s involvement [5, 6]. Thus, more than half of re-partnered mothers formed unions with men with higher economic capabilities than their former partners [7]. On the other hand, the formation of a new family means a further adjustment to changing living conditions for the child, and it may be associated with conflicts of loyalty towards the parent not living in the household or conflicts of rivalry with the parent’s new partner [8]. Psychological studies have shown that the stepparent-stepchild relationship is often not equivalent to a birthparent-child relationship and that stepparents are less altruistic toward their stepchildren than biological parents [9]. However, this observation is shown to be dependent on the availability of socio-economic resources and the living conditions [10].

Overall, transitions from one family structure to another—like the parents’ separation or the new formation as a stepfamily—can be seen as a time of instability, which, for young people, requires considerable coping and adaptation. This is especially the case, if various stressors accumulate.

### Current state of research

At present, there is a large body of studies investigating the impact of family structure on the development of children and adolescents. The focus in these studies has been mostly on wellbeing, behavioural or emotional problems, social development, and academic achievements, while there have been fewer studies on the physical health status of young people. Usually, living with both birth parents is associated with better performance with regard to a variety of social, academic, emotional, behavioural, and health outcomes [4, 11]. Concerning health-related outcomes, many studies have found differences in the wellbeing and health status of children and adolescents, depending on the structure of the family they lived in, although the association seems to be stronger with mental health than with physical health [12].

In most studies, the parental or self-rated general health status of children and adolescents living with both birth parents has been found to be better than in most other family arrangements [12–17].

Adverse outcomes in the mental health status of children not living with both birth parents have been observed for mental wellbeing as well as for behavioural and emotional problems such as depression or anxiety. Compared with nuclear families (children living with both birth parents), emotional and behavioural problems are more prevalent in single parent families [18, 19] as well as in stepfamilies [20, 21].

Children and adolescents living with both birth parents also have a higher health-related quality of life (HRQoL) than those who live in a single parent family [22] or a stepfamily [21].

Regarding adolescents’ health behaviour, most studies have shown that adolescents living with both birth parents smoke less [15, 23–27] and drink less or were engaged less frequently in heavy alcohol use [28–31] than adolescents from other family arrangements. However, a few studies did not find differences in health risk behaviour between family structures [29]. There have been contradictory results as to whether the effects of parental separation and family transitions vary depending on age of the child. Some studies examining the timing of the parents’ separation have found the most harmful effects in early childhood and pre-school age [32] whereas other studies have shown that family instability is more harmful in later childhood than in early childhood [25]. Other studies have suggested that adolescents were particularly vulnerable to parental divorce and family transitions compared with younger children [33]. In reviewing the literature, Amato [34] stated that the majority of findings do not suggest differential effects by children’s age. Likewise, with regard to gender differences in the association of family structure and health no clear conclusions could be drawn, and this seems to be especially the case in adolescence [4, 11, 35–37].

A further time-related research question is whether there are only contemporary adverse health effects associated with the parents’ separation, and if later on there is an adaptation to the new family situation or whether living in a non-nuclear family is associated with some adverse health effects in the long run. There is evidence that successful coping and adaptation take place with decreasing symptoms over time [29], but there are also children and adolescents with disadvantages and downward trajectories over their lifespan [4, 38]. Moreover, there is some empirical evidence for the importance of continuity and stability in family structures, especially for mental health development. For instance, poorer mental health was found in children and adolescents who experienced multiple transitions whereas stable family structure was associated with better health [20, 39], with no or small differences between nuclear families, stable stepfamilies and stable single parent families.

Regarding explanatory mechanisms concerning the impact of family dynamics on adolescents’ health, several accompanying circumstances such as declining socio-economic status (SES) or greater conflict in family relations during or after a family breakdown respectively a stepfamily formation have been discussed [11]. Findings indicated that poorer health-related outcomes among children and adolescents living with single parents were, either totally or partly, a consequence of socio-economic effects [14, 17, 21, 36, 40], whereas in stepfamilies, socio-economic factors did not explain the poorer health status compared with young people in nuclear families [40]. However, not all studies have confirmed these findings [16, 41]. Similarly, parent–child relationships, family connectedness and conflicts within the family can operate as mediators in explaining some of the family structure effects on child wellbeing [33, 42–44].

Another central issue in research on the association between family structure and health in adolescence is whether the associations are causal or driven by selection: Do the family breakdown and/or living in a non-nuclear family impact the health status of young people (causality)? Or can family structure disparities in the health of adolescents be explained by differences in health status before the change of the family composition (selection)? Most of the studies were based on cross-sectional data, which do not allow controlling for health status before any changes in the family status have occurred, so answering this question was not possible. Longitudinal studies that have explicitly addressed causality in analyses of the effects of family dynamics have led to somewhat conflicting conclusions [11]. Many analyses have confirmed that parents’ separation has causal effects, even if they were weaker than the correlations between family structure and outcomes [11, 45], but other studies have reported that these effects result from confounding or selection [44, 46].

Collishaw et al. [47] stressed that there were also important variations in the association between family structure and mental health over time. Whereas the strength of the association between living in a single parent family and conduct problems has not changed during recent decades, in stepfamilies, a significant reduction in the prevalence of conduct problems was found. Therefore, findings from previous studies should be updated by current results.

It also should be noted that most of the above studies were from the United States. Bjarnason et al. [23, 28] found in their international comparative studies that the strength of the association between family structure and health risk behaviour depended to some extent on country-specific values (prevalence of non-nuclear families or societal-level alcohol consumption patterns). Chapple et al. [45] concluded in their meta-analysis that the effect sizes for family structure on health differed across several countries, but it was not possible to link this systematically to differences in policies. Therefore, findings from international studies cannot be simply transferred to German society.

There have been some studies in Germany examining the association between family dynamics and the health status of children and adolescents. We found some studies based on cross-sectional data [15, 17, 21, 48–50], but only a few analyses based on longitudinal data [36, 39, 51].

Regarding the current state of research and relevant issues, we analysed the association between family structure and health/health behaviour in German adolescents based on current data from the KiGGS cohort study. For our analysis, we chose three global health dimensions (self-rated general health, mental health and HRQoL) as well as two health risk behaviours (smoking and heavy episodic drinking) which were discussed as coping strategies in adolescence.

### Research questions

1. Are there differences in general and mental health, HRQoL as well as health risk behaviour of adolescents according to family structure?
2. Is the association between family structure and general and mental health, HRQoL as well as health risk behaviour of adolescents mediated by differences in:
  - SES at baseline or a change in SES between baseline and follow-up,
  - family cohesion at baseline or a change in family cohesion between baseline and follow-up, and/or
  - health status at baseline?

## Materials and methods

### Data and weights

The analysis was conducted using longitudinal data obtained from the first two waves of the German KiGGS cohort study. The “German Health Interview and Examination Survey for Children and Adolescents” (KiGGS) is carried out by the Robert Koch Institute and is part of the health monitoring commissioned by the German Federal Ministry of Health.

The KiGGS baseline study ran from 2003 to 2006 and was realized as a health examination and interview survey. It was the first nationwide representative survey to collect comprehensive health data on children and adolescents aged 0 to 17 years with primary residence in Germany [52]. Participants were enrolled in two steps: first, a systematic sample of 167 primary sample units was drawn from an inventory of German communities (sample points); second, subjects were randomly selected from the official registers of local residents [52]. In total, a response rate of 66.6% with 17,641 respondents was reached (8,985 boys and 8,656 girls). To allow population-based statements for all analyses, a weighting factor was calculated to correct the deviations in the net sample from the population structure (as of 31 December 2004) in terms of age, sex, region, nationality, and parents’ education level [52].

All participants from the baseline study were invited to be part of the first follow-up study called “KiGGS wave 1” conducted as a computer-assisted telephone interviewing between 2009 and 2012. At this time, their ages ranged from 6 to 24 years, with a response rate of 68.5% for the whole KiGGS cohort (n = 11,995) [53]. Differences in nonresponse were partly corrected by a longitudinal weighting factor. In addition to population adjustments, it equates the different probabilities of re-participation in the follow-up study [53]. A comparison of the weighted baseline sample characteristics (t0) of all study participants and those who have re-participated in KiGGS Wave 1 (aged 4 to 12 years) is shown in S1 Table.

Because we focused on the period of adolescence in the present analysis, we included only young people aged 11 to 17 years at time of KiGGS wave 1 data collection. Because of the low prevalence of regular smoking and heavy episodic drinking in the age group of 11 to 13 years (under 3%), we used only data for adolescents aged 14 to 17 years for both health risk behaviours. Response rates within these age groups was 76.0% for 11 to 13 year olds and 72.4% for 14 to 17 year olds [53]. Because the numbers were very small, participants who lived at the households of their grandparents or in institutionalized homes were excluded from this analysis. Additionally, respondents who lived at baseline in a non-nuclear family and in KiGGS wave 1 in a nuclear family were not taken into account here. Participants with missing data for the main residence in one of the two waves were excluded from the analysis, too. The final sample comprised 4,692 respondents in total with 2,629 aged 14 to 17 years.

Unweighted non-response analysis for baseline health indicators as well as for socio-demographic characteristics included in the present study showed differences between children who participated again and those who dropped out. These disparities could be adjusted by the calculated weighting factors [53].

### Variables and measuring instruments

#### Outcome variables

The following indicators of health status and health risk behaviour at follow-up measurement were analysed within the context of family structure and transitions of family structures—all of them reported by the adolescents themselves:

Self-rated health is operated by the first Minimal European Health Module (MEHM1) question. The formulation “In general, what would you say is your health status like?” is based on the recommendations of the World Health Organization (WHO) with a five-step answering scale from very good to very poor. In our statistical analyses, we used the metric variable, where a value of 1 means very good health and 5 corresponds to very poor health.

Emotional and behavioural problems are measured by the self-reports of the “Strengths and Difficulties Questionnaire” (SDQ) [54]. The SDQ is an international established screening instrument measuring strengths and difficulties in mental health. It consists of 25 items that contain different subscales and the possibility to compute the total difficulties score, which was used in this analysis. The total difficulties score includes 20 items referring to emotional symptoms, conduct problems, hyperactivity or inattention and peer relationship problems, with a total range from 0 to 40 with higher values indicating greater difficulties.

HRQoL is measured with the international validated instrument “KIDSCREEN-10” [55]. All item scores were added and transformed into values from 1 to 100, where in the original version higher values indicate better quality of life. For easier comparison between the outcomes, we inverted the scale so that higher values indicate lower HRQoL.

Regular smoking was defined as smoking at least once a week (yes/no).

Heavy episodic drinking was defined based on responses on the three-item screening tool “Alcohol Use Disorders Identification Test” (Audit-C) [56]. The variable indicates heavy episodic drinking (more than five alcoholic beverages per occasion) at least once per month (yes/no).

#### Predictor variable

Data on family status are based on the parent-reported main residence of their child from each wave, operated by the question “With whom does your child live most of the time?”. Children in nuclear families were defined as living together with both biological parents, regardless of whether stepsiblings or half-siblings were also living in the family. Children in single parent families live together with only one parent, whereas stepfamilies were characterized by living together with a biological and a social parent. Transitions of family structure between the two waves were operationalized by computing a new variable with seven categories in total. Three of the categories show stable family structures (stable nuclear, stable single parent, stable stepfamily) and four of them include transitions from one to another family structure (nuclear to single parent family; nuclear to stepfamily; single parent to stepfamily; step to single parent family).

#### Control and mediator variables

To control for confounding, we included the variables age (in full years) and sex. As mediator variables, we used SES, family cohesion as well as the health status at baseline.

SES was defined as a score-index constructed from the level of education, household net income and professional status reported by the parents and having values between 3 (low SES) and 21 points (high SES) [57]. SES at baseline, as well as a variable that comprises the difference between baseline (t0) and follow-up (t1) were included.

Family cohesion was measured with a subscale of the family climate scale by Schneewind et al. [58]. For both waves, the values of the four items were added and transformed into a scale from 0 to 100. Higher score indicate better family cohesion. We used information on the parent-rated scale at baseline. To measure changes in family cohesion between the two waves, we formed a variable that contains the difference between baseline and follow-up. For the follow-up, we used the scale of ratings by the adolescents themselves.

With respect to the health status of the adolescents at baseline, we considered parent-rated general health as well as emotional and behavioural problems, measured by the parent version of the “Strengths and Difficulties Questionnaire” (SDQ) [54]. Regarding the analysed health risk behaviours, smoking, and alcohol consumption, no baseline data were available because of the young age of the participants at the time.

The sample characteristics are shown in Table 1.

**Table 1:** Sample characteristics.

	Mean (SE) weighted	% (95% CI) weighted	n unweighted	Missing n (%) unweighted
**Total**			**4,692**	
**Outcome variables**				
Self-rated health (t1)	1.82 (0.01)		4,692	0
Emotional and behavioural problems (t1)	9.43 (0.08)		4,691	1 (0.0)
Health-related quality of life (t1)	46.74 (0.17)		4,627	65 (1.4)
Regular smoking* (t1)				6 (0.2)
Yes		12.3 (10.6–14.1)	297	
No		87.7 (85.9–89.4)	2,326	
Heavy episodic drinking* (t1)				7 (0.3)
Yes		20.4 (18.2–22.8)	501	
No		79.6 (77.2–81.8)	2,121	
**Predictor variable**				
Family status (t0 -> t1)				0
Nuclear family → nuclear family		76.0 (74.0–78.0)	3,664	
Nuclear family → single parent family		5.4 (4.6–6.3)	282	
Nuclear family → stepfamily		1.8 (1.3–2.3)	89	
Single parent family → single parent family		7.3 (6.2–8.6)	277	
Single parent family → stepfamily		2.7 (2.1–3.5)	109	
Stepfamily → stepfamily		4.9 (4.1–5.9)	203	
Stepfamily → single parent family		1.9 (1.3–2.7)	68	
**Control variables**				
Sex				0
Girls		48.6 (47.1–50.1)	2,311	
Boys		51.4 (49.9–52.9)	2,381	
Age (in full years) (t1)	13.97 (0.03)			0
Age groups (t1)				0
11–13		44.0 (42.4–45.6)	2,063	
14–17		56.0 (54.4–57.6)	2,629	
**Mediator variables**				
Socio-economic status (t0)	11.43 (0.11)		4,684	8 (0.2)
Socio-economic status (t1-t0)	0.51 (0.40)		4,675	17 (0.4)
Family cohesion (t0)	77.03 (0.32)		4,626	66 (1.4)
Family cohesion (t1-t0)	-0.88 (0.43)		4,621	71 (1.5)
Parent-rated general health (t0)	1.65 (0.01)		4,682	10 (0.2)
Parent-rated emotional and behavioural problems (t0)	8.40 (0.10)		4,678	14 (0.3)

* Data on regular smoking and heavy episodic drinking refer only to age groups 14–17 years (n = 2,629).

### Data analysis

Effects of family structure on health status at time of KiGGS wave 1 were calculated by using linear regression models for the three indicators of health status and logistic regression models for the analysis of the two health risk behaviour variables. For each outcome, four different models were calculated, including the mediator variables stepwise: In model 1, we adjusted for age and sex. Baseline SES as well as the change in SES between both time-points were included in model 2. Model 3 additionally considered family cohesion. Afterwards we adjusted for health status at baseline by including parental-rated general health as well as emotional and behavioural problems (model 4). To compare the relative effects of our predictors, which were measured on different scales, we finally z-transformed the fully adjusted model (last column in the tables).

Furthermore, we included interaction terms in our models to investigate if there were sex differences within family structures in relation to each of the health outcomes using the Wald test. As we found no significant sex differences (except for HRQoL), these models are not reported in the tables.

To take clustering of sample points and weighting for the calculation of p values and confidence intervals into account, all analyses were performed with survey procedures (svy) in Stata/SE13 statistical package (StataCorp, College Station, TX, USA). Significance was set at p = 0.05.

## Results

Adolescents who still lived in nuclear families at follow-up, showed the best scores on self-rated health (Table 2). In general, adolescents from families that did not experience any change between baseline and follow-up had better self-rated health than youngsters from families that experienced a transition in this period. Young people who at baseline were living in nuclear families and at follow-up reported living in stepfamilies had the worst self-rated health.

**Table 2:** Mean values / proportions of the health outcomes according to family status.

Family status (Baseline → Follow Up)		Nuclear → Nuclear	Nuclear → Single parent	Nuclear → Step	Single parent → Single parent	Single parent → Step	Step → Step	Step → Single parent	P value
**Self-rated health**	Mean	1.793	1.900	2.034	1.883	1.920	1.873	1.991	0.020
	(SE)	(0.014)	(0.039)	(0.098)	(0.058)	(0.122)	(0.054)	(0.120)	
**Emotional and behavioural problems**	Mean	9.109	10.197	10.551	10.527	10.167	10.459	11.068	<0.001
	(SE)	(0.093)	(0.344)	(0.666)	(0.414)	(0.543)	(0.564)	(0.833)	
**Health-related quality of life**	Mean	46.334	47.956	46.659	48.055	48.748	47.782	49.095	0.008
	(SE)	(0.192)	(0.716)	(0.940)	(0.753)	(1.146)	(0.879)	(1.608)	
Regular Smoking*	%	10.1	20.8	30.3	14.1	16.3	17.7	25.5	0.001
	(95% CI)	(8.5–12.0)	(12.5–32.4)	(12.4–57.0)	(7.5–25.1)	(6.0–37.5)	(9.8–29.9)	(12.5–45.1)	
Heavy episodic drinking*	%	20.2	21.1	24.1	19.3	16.6	26.9	14.5	0.716
	(95% CI)	(17.6–23.1)	(13.8–30.9)	(11.1–44.7)	(13.2–27.3)	(6.2–37.3)	(16.7–40.3)	(6.5–29.4)	

* Regular smoking and heavy episodic drinking were estimated only for age groups 14–17 years.

Regarding emotional and behavioural problems, adolescents living in stable nuclear families reported the fewest problems, while adolescents who at baseline were living in stepfamilies and at follow-up were in single parent families, had the worst scores for emotional and behavioural problems.

Adolescents in stable nuclear families also showed the best HRQoL scores. The worst scores were found in adolescents from families who had transitioned from step to single parent family or from single parent family to stepfamily.

The lowest percentage of regular smokers was found among adolescents in stable nuclear families and stable single parent families. The highest percentage of regularly smoking adolescents was found in families that transitioned from nuclear to stepfamily and from step to single parent family.

For heavy episodic drinking, we found no significant differences with regard to family status.

Table 3 shows the results from the regression models for self-rated health. Adolescents who experienced a change from nuclear to stepfamily reported poorer health compared with youngsters from stable nuclear families. Similar effect was seen in adolescents who had a change from nuclear to a single parent family. However, this effect was not significant when family cohesion was included in the model (models 3 and 4).

**Table 3:** Results from the linear regression models on self-rated health. Adolescents aged 11 to 17 years.

Self-rated health	Model 1		Model 2		Model 3		Model 4		Standardized Coefficients
	Coeff.	P- value	Coeff.	P-Value	Coeff.	P-Value	Coeff.	P-Value	
**Family status (baseline → follow-up)**									
Nuclear → Nuclear	Ref		Ref		Ref		Ref		
Nuclear → Single parent	**0.11**	0.013	**0.09**	0.028	0.04	0.340	0.05	0.285	0.017
Nuclear → Step	**0.24**	0.019	**0.24**	0.020	**0.22**	0.018	**0.22**	0.019	**0.049**
Single parent → Single parent	0.09	0.108	0.07	0.241	0.05	0.387	0.03	0.559	0.013
Single parent → Step	0.12	0.297	0.11	0.330	0.08	0.453	0.06	0.562	0.016
Step → Step	0.09	0.125	0.09	0.128	0.07	0.231	0.05	0.326	0.019
Step → Single parent	0.19	0.115	0.14	0.241	0.12	0.307	0.16	0.150	0.035
Sex: female	**0.07**	0.003	**0.07**	0.004	**0.06**	0.011	**0.07**	0.001	**0.060**
Age (t1)	0.00	0.664	0.00	0.470	-0.01	0.251	-0.01	0.187	-0.027
Socio-economic status (t0)			**-0.02**	0.000	**-0.01**	0.000	**-0.01**	0.007	**-0.061**
Socio-economic status (t1-t0)			**-0.01**	0.028	-0.01	0.080	-0.01	0.146	-0.029
Family cohesion (t0)					**-0.01**	0.000	**-0.01**	0.000	**-0.181**
Family cohesion (t1-t0)					**-0.01**	0.000	**-0.01**	0.000	**-0.248**
Parent-rated health (t0)							**0.12**	0.000	**0.116**
Emotional & behavioural problems (t0)							**0.01**	0.016	**0.065**

Range of values: 1 (very good health)– 5 (very poor health).

Regarding emotional and behavioural problems, we found higher coefficients for adolescents across all family statuses compared with adolescents continuously living in a nuclear family (Table 4) although not all of them reached statistical significance. Significant coefficients were visible for all adolescents who lived in a single parent family at follow-up, regardless of the family status at baseline, as well as for adolescents who lived in a stepfamily continuously. When controlling for SES, adolescents who experienced a transition from a stepfamily to a single parent family no longer showed significantly more emotional and behavioural problems (model 2), while including family cohesion and health at baseline led again to a significant higher coefficient in this family structure (models 3 and 4). When including family cohesion in the model, the coefficients for all family statuses decreased. This was especially the case for adolescents who experienced a transition from nuclear to single parent family. Controlling for health status at baseline again lowered the effect of the family status on emotional and behavioural problems. Thus, in the fully adjusted model, we observed a significantly higher score only among adolescents from families who had transitioned from a stepfamily to a single parent family.

**Table 4:** Results from the linear regression models on emotional and behavioural problems (SDQ total score). Adolescents aged 11 to 17 years.

Emotional and behavioural problems	Model 1		Model 2		Model 3		Model 4		Standardized Coefficients
	Coeff.	P- value	Coeff.	P-Value	Coeff.	P-Value	Coeff.	P-Value	
**Family status (baseline → follow-up)**									
Nuclear → Nuclear	Ref		Ref		Ref		Ref		
Nuclear → Single parent	**1.07**	0.004	**1.05**	0.004	0.34	0.282	0.38	0.232	0.019
Nuclear → Step	1.35	0.057	1.33	0.060	1.08	0.099	1.15	0.078	0.034
Single parent → Single parent	**1.47**	0.001	**1.17**	0.006	**0.98**	0.015	0.66	0.069	0.039
Single parent → Step	1.01	0.050	0.82	0.115	0.43	0.363	0.10	0.829	0.004
Step → Step	**1.49**	0.010	**1.43**	0.012	**1.19**	0.019	0.73	0.156	0.035
Step → Single parent	**1.92**	0.018	1.45	0.069	**1.11**	0.049	**1.08**	0.029	**0.032**
Sex: female	**0.91**	0.000	**0.90**	0.000	**0.76**	0.000	**1.02**	0.000	**0.113**
Age (t1)	**-0.15**	0.001	**-0.17**	0.000	**-0.24**	0.000	**-0.24**	0.000	**-0.105**
Socio-economic status (t0)			**-0.16**	0.000	**-0.15**	0.000	**-0.08**	0.000	**-0.070**
Socio-economic status (t1-t0)			-0.06	0.171	-0.05	0.196	-0.02	0.594	-0.011
Family cohesion (t0)					**-0.11**	0.000	**-0.09**	0.000	**-0.291**
Family cohesion (t1-t0)					**-0.10**	0.000	**-0.10**	0.000	**-0.417**
Parent-rated health (t0)							0.07	0.630	0.010
Emotional & behavioural problems (t0)							**0.19**	0.000	**0.227**

Range of values: 0 (low problems)– 40 (strong problems).

In comparison to the reference group, we observed higher coefficients for low HRQoL in all non-nuclear family statuses, although not all differences were statistically significant. Adolescents who experienced transition from nuclear to single parent family, from single parent to stepfamily, or were in a stable single parent family reported significantly worse HRQoL than adolescents who remained in stable nuclear families (Table 5). However, the significance of these relations disappeared after controlling for family cohesion and health status at baseline.

**Table 5:** Results from the linear regression models on health-related quality of life. Adolescents aged 11 to 17 years.

Health-related quality of life	Model 1		Model 2		Model 3		Model 4		Standardized Coefficients
	Coeff.	P- value	Coeff.	P-Value	Coeff.	P-Value	Coeff.	P-Value	
**Family status (baseline → follow-up)**									
Nuclear → Nuclear	Ref		Ref		Ref		Ref		
Nuclear → Single parent	**1.58**	0.034	1.50	0.052	0.22	0.739	0.27	0.672	0.007
Nuclear → Step	0.82	0.422	0.81	0.427	0.31	0.777	0.38	0.738	0.006
Single parent → Single parent	**1.58**	0.036	**1.49**	0.048	1.05	0.151	0.75	0.293	0.023
Single parent → Step	**2.33**	0.021	**2.29**	0.022	1.41	0.092	1.12	0.163	0.022
Step → Step	1.33	0.137	1.36	0.131	0.83	0.304	0.43	0.602	0.011
Step → Single parent	2.33	0.111	2.11	0.145	1.69	0.126	1.43	0.226	0.022
Sex: female	**2.52**	0.000	**2.50**	0.000	**2.22**	0.000	**2.47**	0.000	**0.144**
Age (t1)	**0.76**	0.000	**0.74**	0.000	**0.60**	0.000	**0.60**	0.000	**0.140**
Socio-economic status (t0)			-0.08	0.081	-0.05	0.188	0.01	0.773	0.005
Socio-economic status (t1-t0)			-0.08	0.277	-0.07	0.270	-0.04	0.508	-0.012
Family cohesion (t0)					**-0.22**	0.000	**-0.19**	0.000	**-0.336**
Family cohesion (t1-t0)					**-0.21**	0.000	**-0.21**	0.000	**-0.482**
Parent-rated health (t0)							0.33	0.183	0.023
Emotional & behavioural problems (t0)							**0.17**	0.000	**0.107**

Range of values: 1 (high HRQoL)– 100 (low HRQoL).

The interaction between sex and family status was significant for the case of HRQoL. Sex-stratified analysis showed that, in comparison to boys and girls in stable nuclear families, in boys, the transition from nuclear to stepfamily was associated with lower HRQoL. On the other hand, the transition from a stepfamily to a single parent family and from single parent to a stepfamily was only problematic for girls (results shown in S2 Table).

Adolescents who experienced changes from nuclear either to step or single parent family or from step to single parent family had significantly higher odds of smoking compared with adolescents from stable nuclear families (Table 6). This association remained significant after controlling for all mediator variables in the model.

**Table 6:** Results from the logistic regression models on regular smoking. Adolescents aged 14 to 17 years.

Regular Smoking	Model 1		Model 2		Model 3		Model 4		Standardized Coefficients
	OR	P- value	OR	P-Value	OR	P-Value	OR	P-Value	
**Family status (baseline → follow-up)**									
Nuclear → Nuclear	Ref		Ref		Ref		Ref		
Nuclear → Single parent	**2.46**	0.005	**2.40**	0.005	**2.10**	0.015	**2.09**	0.020	**1.184**
Nuclear → Step	**5.69**	0.021	**5.74**	0.016	**4.94**	0.021	**5.61**	0.012	**1.222**
Single parent → Single parent	1.35	0.408	1.24	0.551	1.10	0.790	0.95	0.880	0.987
Single parent → Step	1.76	0.327	1.71	0.355	1.62	0.401	1.56	0.439	1.073
Step → Step	2.03	0.063	2.01	0.070	1.90	0.107	1.69	0.168	1.134
Step → Single parent	**3.95**	0.006	**3.41**	0.016	**3.24**	0.026	**3.24**	0.024	**1.190**
Sex: female	0.89	0.509	0.88	0.477	0.84	0.347	0.94	0.724	0.968
Age (t1)	**1.97**	0.000	**1.97**	0.000	**1.95**	0.000	**1.99**	0.000	**2.200**
Socio-economic status (t0)			**0.95**	0.026	**0.95**	0.032	0.97	0.294	0.905
Socio-economic status (t1-t0)			0.97	0.490	0.97	0.498	0.98	0.663	0.963
Family cohesion (t0)					**0.98**	0.000	0.99	0.075	0.847
Family cohesion (t1-t0)					**0.99**	0.009	**0.99**	0.007	**0.777**
Parent-rated health (t0)							0.97	0.820	0.980
Emotional & behavioural problems (t0)							**1.08**	0.000	**1.505**

None of the family structures showed any significant association with heavy episodic drinking (Table 7).

**Table 7:** Results from the logistic regression models on heavy episodic drinking. Adolescents aged 14 to 17 years.

Heavy episodic drinking	Model 1		Model 2		Model 3		Model 4		Standardized Coefficients
	OR	P- value	OR	P-Value	OR	P-Value	OR	P-Value	
**Family status (baseline → follow-up)**									
Nuclear → Nuclear	Ref		Ref		Ref		Ref		
Nuclear → Single parent	1.08	0.790	1.08	0.786	0.95	0.864	0.94	0.838	0.986
Nuclear → Step	1.70	0.313	1.69	0.312	1.51	0.406	1.51	0.399	1.049
Single parent → Single parent	0.83	0.488	0.82	0.469	0.73	0.278	0.74	0.289	0.925
Single parent → Step	0.76	0.638	0.75	0.632	0.70	0.540	0.71	0.541	0.947
Step → Step	1.54	0.212	1.51	0.242	1.43	0.310	1.40	0.339	1.083
Step → Single parent	0.78	0.577	0.77	0.560	0.70	0.450	0.74	0.527	0.956
Sex: female	**0.63**	0.000	**0.63**	0.000	**0.62**	0.001	**0.61**	0.001	**0.781**
Age (t1)	**1.88**	0.000	**1.87**	0.000	**1.89**	0.000	**1.91**	0.000	**2.100**
Socio-economic status (t0)			0.99	0.785	0.99	0.575	0.98	0.430	0.940
Socio-economic status (t1-t0)			1.00	0.970	1.00	0.998	1.00	0.938	0.995
Family cohesion (t0)					**0.98**	0.002	**0.98**	0.002	**0.774**
Family cohesion (t1-t0)					**0.99**	0.021	**0.99**	0.022	**0.820**
Parent-rated health (t0)							0.83	0.142	0.896
Emotional & behavioural problems (t0)							1.00	0.884	0.989

## Discussion

The aim of this analysis was to investigate the association between family structure and different health-related outcomes in adolescence. Consistent with the current state of research, we found adolescents who continuously lived with both birth parents to be in good health. However, not all non-nuclear families were associated with adverse health outcomes. There were diverse patterns according to the outcome studied. When the parents’ separation took place after the baseline survey, young people reported poorer health and were more likely to smoke. The transition from stepfamily to single parent family was also associated with a higher risk of regular smoking. Lower HRQoL as well as higher scores for emotional and behavioural problems were found in almost all non-nuclear family structures, although not all effects reached statistical significance—this may be due to the small sample size in some family subgroups. Although many international studies confirmed the association between family structure and higher alcohol consumption [24, 28, 30, 31], we found no higher rates of heavy episodic drinking in adolescents living in non-nuclear families. We assume that according to family-related factors, parental drinking behaviour (e.g. exposure to drunkenness within the family [30]) as well as parent–child relations [59] may play a greater role than family structure per se.

We also examined whether and to which extent SES and family cohesion explain family structure disparities. The contribution of family cohesion reducing the estimated effects for the family subgroups was much higher than the contribution of SES. Regarding emotional and behavioural problems as well as HRQoL, this was the case for all subgroups. According to self-rated health status we saw a reduction of the effect size only in adolescents transitioning from nuclear to single parent families. Thus, the association between family structure and mental health outcomes in particular was explained to a relatively large extent by family cohesion. Obviously, the quality of the familial relationship plays an important mediating role. This is consistent with international research [33, 42, 44]. For example, Cavanagh [42] showed that the effects of family structure on emotional distress and drug use were explained by differences in the quality of family relationships such as parent–adolescent closeness and family connectedness. Sun [44] found that wellbeing deficits among adolescents in post-disruption families could be largely predicted by family circumstances (such as less intimate parent–child relationship) even before and during the period coinciding with the family breakdown. However, in the study of Pálmarsdóttir [43] family conflict did not fully mediate the effects of divorce on depression in adolescence. Nevertheless, it must be considered that it is not clear whether the measured difference in family cohesion between both waves actually reflected a change in the family relationships, or whether the difference in the scores was due to disparities in the assessments of parents (baseline) and adolescents (follow-up).

In contrast, SES functioned not at all or only to a small extent as a mediator of the association between family structure and adolescents’ health and health risk behaviour. Only emotional and behavioural problems in adolescents experiencing a transition from step to single parent family were found to be mediated by SES. We had assumed that SES would be an important mediator in the health of adolescents in single parent families as has been shown in some studies [40]. This hypothesis was not confirmed in our analysis. Other studies also found that SES only partially [16] or did not at all [41] explain family structure disparities in adolescents’ wellbeing.

The question of whether family structure disparities in health indicators are shaped by causation or selection effects is an important issue in empirical research on associations between family structure and adolescents’ health. To examine this topic, we included health status at baseline in the analysis and found it to play no significant mediating role for family structure disparities in adolescents’ health risk behaviours and self-rated health. In contrast, according to family structure disparities in adolescents’ emotional and behavioural problems the health status at baseline had a strong mediating effect. To a smaller extent, this was observed for HRQoL as well. This means that differences in mental health according to family structure were partly explained by the pre-transition health status. One possible mechanism could be that poor pre-transition health outcomes in children—especially if these are emotional or behavioural problems—may cause or increase parents’ or stepparents’ stress which increases the likelihood of separation. Another possible underlying mechanism is that poor pre-transition health outcomes in children may be the result of partner conflict within the family that leads to separation or divorce and which is the ultimate cause of the poor outcomes, rather than family structure per se [45]. Our results confirm that failing to control for pre-transition outcomes can result in an over-estimation of the impact of family transitions because of selection effect [45]. An alternative explanation is that there might be a familial hereditary predisposition for unstable romantic relationships, similar to health problems. For example, Liu et al. showed that love-related behaviours are associated with serotonin levels in the brain [60].

In the fully adjusted models, we found very small disparities according to family structure for all outcomes—except smoking. Regarding regular smoking, no significant changes in the effect sizes of family structures were detected by controlling for SES, family cohesion and health status at baseline. This could be interpreted to indicate that smoking is an effect of certain family transitions (especially the separation of the birth parents and the transition from step to single parent family). Thus, smoking may be a strategy in adolescence to cope with family instability. Another explanation could be lower parental monitoring during a period of family instability [27, 33].

To summarize, we found two high-risk groups: adolescents who had experienced parental separation within the last 6 years, as well as adolescents whose families transitioned from a stepfamily to a single parent family during this time period. The last group is characterized presumably by the most experienced family structure transitions, although we have no exact information about the number of family transitions. Fomby et al. [61] have found evidence of the hypothesis that family instability in particular (measured by the numbers of transitions) is associated with poorer outcomes, although Lee and McLanahan [62] postulated that the type of family transition is more important for the development of children than the numbers of transitions.

Considering the substantial differences in existing studies regarding study design, analytic strategies, outcomes and the age ranges of the children and adolescents included in the analysis, overall, our results were largely in accordance with the current state of research. In sum, the differences in health status and health risk behaviour of adolescents according to family structure seemed to be relatively small. In all family structures, most adolescents were in good health and did not behave in a seriously risky way. Causal effects of family structure on adolescents’ health in a strict sense (controlled for pre-transition health) were even lower. Chapple et al. [45] in their meta-analysis came to the conclusion that the better the quality of the study, the smaller is the effect size found.

### Strength and limitations

The strength of our study was the use of the follow-up of a representative sample (in terms of age, sex, region, nationality, and parents’ education level) of German adolescents. The large sample size allowed us to analyse small family subgroups. Furthermore, the KiGGS cohort study comprised a wide variety of different health-related outcomes and social determinants and is, therefore, unique to Germany.

Although the KiGGS study applied numerous strategies to improve the response-rate of children and adolescents who are hard to reach, the possibility of a selection bias (at the stage of selecting participants for KiGGS baseline (t0) or loss to follow-up (t1)) cannot be completely ruled out. Perhaps, after a family transition and a sub-sequent relocation, families might be re-contacted with more difficulty than those who stayed put at follow-up. This must be taken into account when interpreting the results. However, because of the very strict data protection regulations in Germany, no routine data could be used for the analysis. Furthermore, the aim of this paper was not to report representative prevalence of health outcomes, but to analyse the association of family structure and health. Another limitation is that we only had data from two survey waves and that the period between the waves was quite long (6 years). Moreover, we had only information on family composition at the time of data collection. We have no information on whether other transitions took place in the periods before the baseline survey or between the surveys. Regarding the transition from nuclear to stepfamily, it could be assumed that in the 6-year-period between the surveys there was at least one additional transition in most cases. We also had no data available according to the youngsters’ age at the time of family transitions, especially the separation of birth parents. Furthermore, it cannot be ruled out that health impairments in adolescents after a family transition occur later in development and, hence, could not be measured here. We are aware that the results presented here allow no final assessment regarding the effect of family transitions on adolescent’s health in terms of causality and selection. Another important mediator mentioned in the research literature is parental mental health status, which we did not adjust for in our analysis. Information on other factors possibly mediating the association of family structure and health could not be included in the analysis because data for these indicators were not collected in the KiGGS study. Among these were parental conflicts (especially in the phase of separation), the quality of relationship with the custodial parent, the non-resident birth parent or the stepparent, the frequency of contact with the non-resident parent, parenting style, and the social embeddedness of the family.

Because of the small sample sizes in some familial subgroups we carried out our analysis without stratifying by adolescents’ sex. However, we proved sex differences by calculating interaction effects between family status and sex. For all outcomes—except HRQoL—we found no significant moderating effect of adolescents’ sex.

The analysis is based on parents’ and adolescents’ self-reports; no objectively measured indicators were included in this analysis. For the baseline, we used data collected only from the parents as proxies because self-reports from the youngsters were collected only from the age of 11 years and, thus, were not available for the age group examined here. It is important to keep in mind that differences in health according to family structure could be influenced by a possibly greater sensitivity to psychosocial stress in adolescents after a family breakdown or the new formation of a stepfamily.

Because the survey method was changed between the baseline survey and KiGGS wave 1, method effects could not be excluded fully. Regarding the family structure, we presumed only very small method effects because the recording of the household composition could be evaluated as a robust and well established survey instrument.

### Further research

With completion of data collection for KiGGS wave 2 in 2017 [63] we will have further data on the age of the participants at the time of parents’ separation as well as on parenting style, parental mental health and other adverse childhood experiences. Additionally, we will be able to identify blended families. Thus, in-depth causal analysis regarding the effect of family transition on health will be possible with these data (e.g., using fixed effects and random effects models). Moreover, we plan to analyse how family structure in childhood and adolescence affects the transition into young adulthood and health in this stage of life.

## Conclusions

Although family structure has been shown to have only a moderate direct effect on adolescents’ health when adjusting for family cohesion, SES, and pre-transition health status, the family structure helps to identify adolescents who are at risk [35]. Because family cohesion was found to be an important mediator in the association between family structure and adolescents’ health, prevention programmes as well as interventions, however, should be directed toward the parent–adolescent relationship rather than just the family structure [35]. To minimize the psychosocial stress of young people during periods of the family transition, counselling and mediation programmes may help to sensitize mothers and fathers to the child’s needs and to enable adolescents to process the transition and thus allow parents and their children to remain in good contact.

## Supporting information

S1 Table Comparison of baseline sample characteristics (t0) of all KiGGS baseline responders and KiGGS Wave 1 responders aged 4 to 12 years (weighted proportions or means). (PDF)

S2 Table Results from the linear regression models on health-related quality of life with interaction between family status and sex. Adolescents aged 11 to 17 years.
(PDF)
