# Supplementary material for: Health and health risk behaviour of adolescents—Differences according to family structure. Results of the German KiGGS cohort study
Source: PLoS One. 2018 Mar 7;13(3):e0192968. doi: 10.1371/journal.pone.0192968 (PMC5841741; doi:10.1371/journal.pone.0192968)
Supplement: S1 Table — (PDF) [file pone.0192968.s001.pdf]

S1 Table. Comparison of baseline sample characteristics (t0) of all KiGGS baseline responders and KiGGS Wave 1 responders aged 4 to 12 years (weighted proportions or means).*

	**All KiGGS baseline-responder**[1] n=10,902	**KiGGS Wave 1-responder**[2] n=4,756
	**% (95% CI)**	**% (95% CI)**
Sex (t0)		
Girls	48.7 (48.2-49.2)	48.5 (47.0-50.0)
Boys	51.3 (50.8-51.8)	51.5 (50.0-53.0)
Family form (t0)		
Nuclear family	81.9 (80.5-83.1)	81.7 (79.6-83.6)
Single parent family	11.3 (10.3-12.4)	11.0 (9.5-12.7)
Stepfamily	6.9 (6.3-7.5)	7.3 (6.2-8.5)
	**Mean (SE)**	**Mean (SE)**
Family cohesion (t0)	77.00 (0.20)	76.94 (0.32)
SES (t0)	11.02 (0.09)	11.38 (0.11)
Parent-rated general health (t0)	1.69 (0.01)	1.65 (0.01)
Parent-rated emotional and behavioural problems (t0)	8.74 (0.07)	8.48 (0.11)

* KiGGS Wave 1 population: re-participants who were aged 4 to 12 at baseline; not fully equivalent with the study sample used in this analysis, which comprised only participants aged 11 to 17 years at t1.

[1] Cross-sectional weighting factor for the baseline survey (population as of 31 December, 2004).

[2] Longitudinal weighting factor to compensate for the probability of re-participation and to adjust to the population level at the time of the baseline survey (31 December, 2004).
