# Supplementary material for: Health and health risk behaviour of adolescents—Differences according to family structure. Results of the German KiGGS cohort study
Source: PLoS One. 2018 Mar 7;13(3):e0192968. doi: 10.1371/journal.pone.0192968 (PMC5841741; doi:10.1371/journal.pone.0192968)
Supplement: S2 Table — Adolescents aged 11 to 17 years. (PDF) [file pone.0192968.s002.pdf]

S2 Table. Results from the linear regression models on health-related quality of life with interaction between family status and sex. Adolescents aged 11 to 17 years.

Health-related quality of life	Model 1		Model 2		Model 3		Model 4		Standardized Coefficients
	Coeff.	P-value	Coeff.	P-Value	Coeff.	P-Value	Coeff.	P-Value	
**Family status (baseline → follow-up)**									
Nuclear → Nuclear	Ref		Ref		Ref		Ref		
Nuclear → Single parent	1.33	0.148	1.25	0.180	0.40	0.645	0.42	0.624	0.011
Nuclear → Step	2.71	0.024	2.71	0.025	2.87	0.029	2.89	0.035	0.045
Single parent → Single parent	0.95	0.312	0.88	0.345	0.38	0.676	0.13	0.877	0.004
Single parent → Step	0.05	0.955	-0.01	0.992	-0.40	0.643	-0.61	0.482	-0.012
Step → Step	0.74	0.524	0.73	0.529	0.24	0.793	-0.20	0.837	-0.005
Step → Single parent	-0.72	0.562	-0.93	0.463	0.48	0.655	0.13	0.914	0.002
**Interaction between family status and sex**									
Nuclear → Nuclear # female	Ref		Ref		Ref		Ref		
Nuclear → Single parent # female	0.51	0.715	0.52	0.706	-0.35	0.775	-0.29	0.807	-0.006
Nuclear → Step # female	-3,93	0.062	-3,95	0.061	-5.32	0.014	-5.24	0.019	-0.057
Single parent → Single parent # female	1.32	0.303	1.27	0.322	1.40	0.271	1.30	0.305	0.028
Single parent → Step # female	4.39	0.025	4.42	0.023	3.48	0.042	3.33	0.042	0.047
Step → Step # female	1.38	0.348	1.46	0.323	1.39	0.319	1.50	0.275	0.025
Step → Single parent # female	5.49	0.021	5.47	0.021	2.14	0.274	2.18	0.286	0.026
Sex: female	2.19	0.000	2.17	0.000	2.03	0.000	2.28	0.000	0.133
Age (t1)	0.77	0.000	0.75	0.000	0.61	0.000	0.61	0.000	0.142
Socio-economic status (t0)			-0.08	0.071	-0.06	0.166	0.01	0.826	0.004
Socio-economic status (t1-t0)			-0.08	0.300	-0.07	0.266	-0.04	0.507	-0.012
Family cohesion (t0)					-0.22	0.000	-0.19	0.000	-0.336
Family cohesion (t1-t0)					-0.21	0.000	-0.21	0.000	-0.482
Parent-rated health (t0)							0.33	0.178	0.023
Emotional & behavioural problems (t0)							0.17	0.000	0.106

Range of values: 1 (high HRQoL) – 100 (low HRQoL)
